# Supplementary material for: Consciousness & Brain Functional Complexity in Propofol Anaesthesia
Source: Sci Rep. 2020 Jan 23;10:1018. doi: 10.1038/s41598-020-57695-3 (PMC6978464; doi:10.1038/s41598-020-57695-3)
Supplement: Supplementary file 1 — Supplementary Material Title Page. [file 41598_2020_57695_MOESM1_ESM.pdf]

Consciousness & Brain Functional Complexity in Propofol Anaesthesia: Supplementary Data

Thomas F Varley, Andrea I Luppi, Ioannis Pappas, Lorina Naci, Ram Adapa, Adrian M Owen, David K Menon, Emmanuel A Stamatakis

Supplementary Dataset A

CSV file containing the results of all tests, for each subject in Dataset A, comprising Awake, Mild, and Moderate Propofol Sedation as well as the serum propofol concentrations.

Supplementary Dataset B

CSV file containing the results for all tests, for each subject in Dataset B, comprising Awake and Deep Propofol sedation.
